# Intervention initiatives to raise young people’s interest and participation in STEM

**DOI:** 10.3389/fpsyg.2022.960327

**Published:** 2022-11-18

**Authors:** Barbara Schneider, I-Chien Chen, Lydia Bradford, Kayla Bartz

**Affiliations:** College of Education, Michigan State University, East Lansing, MI, United States

**Keywords:** engagement, social and emotional learning, science learning, interventions, project-based learning

## Abstract

For nearly a decade, two science interventions anchored in project-based learning (PBL) principles have been shown to increase student science learning in 3^rd^ grade and high school physical science classes. Both interventions employed a randomized control trial of several thousand students (N = 3,271 in 3^rd^ grade and N = 4,238 in 10^th^, 11^th^, and 12^th^ grades). Incorporating a rich background of research studies and reports, the two interventions are based on the ideas of PBL as well as the National Academies of Science’s publications, including how children learn; how science learning and instruction can be transformed; and the performance expectations for science learning articulated in the Next Generation of Science Standards. Results show significant positive increases in student academic, social, and emotional learning in both elementary and secondary school. These findings can be traced, in part, to carefully crafted experiential participatory activities and high-quality instructional materials which act as strong facilitators for knowledge acquisition and use. Reviewing the innovations undertaken by these two interventions, this article describes the importance of studying social and emotional factors ‘*in situ*’, using the Experience Sampling Method (ESM), that can motivate and engage students in science learning in both elementary and secondary school. Using these ‘*in situ*’ data collection (N = 596 students in 3^rd^ and N = 1412 students in 10^th^, 11^th^, and 12^th^ grades) along with case studies and repeated measures analysis gave deep insights into emotional and social development for young children and adolescents. These methods should continue to be considered when trying to understand key factors of improving engagement in science.

## Introduction

National and international assessments indicate that US students’ academic performance in science is barely reaching average scores, especially in junior and senior high school (National Assessment of Educational Progress, 2021; Organisation for Economic Co-operation and Development, 2020). More disconcerting is that among certain segments of the US student population performance scores continue to lag behind most students in the general population. Additionally, the stagnant or marginal declining scores of 4^th^ graders on NAEP in 2019 and no changes in the scores of 12^th^ graders affirms the view of researchers, business community, and public stakeholders that US students are unprepared to meet the technological changes of today and likely to have difficulty finding stable employment as adults (see Hammerstein et al., 2021).

These less than promising science achievement test results were evident before the SARS-CoV-2 pandemic. The latest projections, especially among those with the most limited economic and social resources, is that these students are likely to experience major academic, social, and emotional problems at school this coming year and perhaps throughout their careers and beyond (Dorn et al., 2020). The pandemic has raised multiple questions about the long-term effects on student lives and their resilience, having experienced an unprecedented global health crisis. One possible solution for ameliorating these long-term effects is implementing new promising interventions with innovative instructional strategies and materials which show results of increasing science achievement as well as the social and emotional needs of children and adolescents.

For the past several years, two science curriculum interventions have been implemented and evaluated in elementary and secondary schools (Schneider et al., 2022; Krajcik et al., in press). These two grade level interventions share a theoretical design-based rationale, based on project-based learning (PBL) principles, and provide solutions to several serious questions that have been raised about the quality of science instruction in the US. The elementary school intervention, Multiple Literacies in Project-based Learning (ML-PBL), is an efficacy study of 3^rd^ graders in Michigan where students were given four science units in which learning goals were developed consistent with the Next Generation of Science Standards and the instructional experiences were based on the components of three-dimensional learning (disciplinary core ideas, science and engineering practices, and cross-cutting concepts; National Research Council, 2012; Krajcik et al., 2021). The high school study, Crafting Engaging Science Environments (CESE), developed three units in chemistry and three in physics, and was similarly created using the PBL principles, the NGSS performance expectations, and the National Research Council’s definition of three-dimensional learning (Schneider et al., 2022). These units, at the elementary and secondary level, were all designed with experiences to promote students asking questions, collaborating with one another, constructing evidence and artifacts, and engaging in scientific and engineering practices.

One key, new addition found in the ML-PBL and CESE interventions was the explicit importance placed on social and emotional learning and its relationship to science achievement (see chapter by Krajcik and Schneider, 2021 for ML-PBL; see Schneider et al., 2020 for CESE). PBL has implicitly emphasized social and emotional learning with its activities, materials, and assessments that have been deliberately designed to create equitable environments (Miller and Krajcik, 2019). In PBL classrooms all students are encouraged and supported to participate in asking questions, collaborate and work in teams, and share personal science experiences both in- and out-of-the classroom. However, in these two interventions, these ideas were further articulated theoretically and applied with specific methods and items developed to measure the impact of social and emotional factors on science achievement at the elementary and secondary levels. At the elementary level, students were asked questions about their interest, skills, and challenge in specific science activities (Bartz et al., 2022) and these same measures were asked to the secondary students with age-appropriate language (Schneider et al., 2016; Bradford and Bartz, 2022). Additionally, during teacher professional learning sessions, special activities were designed to guide teachers in fostering greater participation and inclusivity among all students (Krajcik and Schneider, 2021; Schneider et al., 2022).

Several considerations in the design of the interventions were identified for understanding social and emotional learning for both elementary and secondary students. First, and most importantly, was the selection of social and emotional constructs that were appropriate for science learning in classrooms (Baines et al., 2017; Lee et al., 2019). Care was taken not to include the entire corpus of psychological measures of emotionality but rather concepts that could be observed (i.e., self-reflection, ownership, and collaboration) and assessed during science lessons. Second, because the focus was on promoting engagement in science learning, fundamental concepts identified in earlier studies of engagement were used to measure interest, skills, and challenge when involved in learning activities (Salmela-Aro et al., 2016; Schneider et al., 2016; Moeller et al., 2017).

For purposes of measurement at the elementary level, social and emotional learning states (i.e., patterns of feelings during activities within specific time periods) were assessed when students were in their science classes. This was a one-time measure, validated through a variety of statistical procedures (see, Krajcik et al., 2021). At the high school level, these constructs were measured ‘*in situ*’ when students who were participating in PBL experiences multiple times during the semester, were randomly notified and asked to answer a survey on their emotionality with the Experience Sampling Method (ESM). The ESM is a type of time diary which uses repeated measures randomly obtained through an intermediate notification system (such as on a phone). The results from these two important additions to the PBL design showed significant positive impacts on science learning, motivation, and engagement. This chapter describes why social and emotional learning is an essential component for academic learning, how we incorporated them in these two different efficacy studies, and how we plan to evaluate their impact on science learning.

## Why we need to care about social and emotional learning

More recently, there has been increased attention within the psychological community to investigate the relationship between the impact of social and emotional learning on student performance in classrooms. Previously, these issues were rarely isolated to the learning context or used to direct teachers’ practices in their classrooms for the purpose of supporting all students’ academic performance and well-being (Durlak et al., 2015; Jagers et al., 2018; Lee et al., 2019). This increased interest in contextualized social and emotional learning support has now been expanded in multiple frameworks to include sensitivity to differences in students’ cultures, equity practices to encourage student participation in classroom experiences, and opportunities for enhanced collaborative and team activities [more specifically as discussed in Lee et al., 2019 and National Academies of Sciences, 2021].

The intentionality of inclusionary social and emotional learning opportunities in classrooms complements PBL principles (Peele-Eady and Moje, 2020; Rosado-May et al., 2020) and the execution in the design of ML-PBL and CESE interventions. One of the most critical aspects of PBL is beginning with a “driving question,” a real-world problem, where students are encouraged and supported to ask meaningful questions that personalize the lesson to their own lives. Based on the driving question, subsequent experiences are enacted whereby students work together finding solutions to these problems over the course of a unit. The significance of the driving question is critical for motivating interest from the perspective of the students’ lives, shaped by their familial and community economic, social, and cultural resources, and forging them on a path of personalized scientific inquiry and discovery. One cannot overlook the fundamental value of beginning science lessons from the standpoint of appealing to the personal interest of the students for “why” pursuing a recognizable puzzling phenomenon in their natural world may have importance to them (Renninger and Hidi, 2020). Results show that personalized meaningful interest in a topic motivates sustained interest in other science learning experiences providing that they are reasonable for the students’ skill sets and are challenging solvable problems. By incorporating these ideas, students are more likely to persist and learn phenomena they may have previously considered unsolvable (Csikszentmihalyi and Schneider, 2000).

The ML-PBL and CESE interventions included carefully crafted lessons which are planned with a series of intra- and inter-connected experiences which coherently increase in scientific knowledge and practices (Fortus and Krajcik, 2012). Lessons are constructed so students complete them with their classmates or individually extend their competencies in planning investigations, building models, and writing scientific explanations, all of which offer support for learning how to formulate evidential claims to problems. Activities typically focus on “hands on” experiences, most often in groups, bringing together students of initially varying ability to have the opportunity to acquire actual scientific skills. These learning experiences are quite different from traditional science instruction which tends to rely on science content that students have to memorize, frequently measured individually with summative tests, and which frustrates many students contributing to the loss of interest in science (National Research Council, 2012; National Academies of Sciences, 2021). Rather, these sequential learning experiences are designed to challenge students to work on problems to which they do not know the answer and to encourage them to continue trying to solve them. These activities, which push students to seek the answer to challenging questions, while doing something important to them, have been shown to be related to feelings of determination (Bradford and Bartz, 2022).

Concentrating on several of the most important social and emotional learning measures, these two interventions also underscored the importance of obtaining such information on these constructs when students are in their science classes. This led in both intervention studies to several assumptions regarding social and emotional measurement: (1) SEL is not a distinctive single psychological state, one can be engaged and feel successful and in control while also feeling a sense of stress; (2) SEL is time variant, in that a confluence of SEL states vary in intensity across the course of one’s daily life experiences; and (3) SEL is highly susceptible to contextual environmental conditions such as the instructional activities in the classroom.

Recognizing developmental differences in literacy, social and emotional awareness of self and others, and technological skills (Lerner and Steinberg, 2009), the selection of SEL measures and the methodology used in the elementary and secondary intervention studies varied in form, rapidity, and replication. However, what they shared is an overlap of SEL states that examined interest, sense of self-appraisal of one’s involvement in specific activities, value of one’s accomplishments, and collaboration with one another. The elementary design was to measure SEL during their science classes. The secondary school study examined moment to moment ‘*in situ*’ experiences of when students were both within and outside their science classes which allowed for the measuring of variations in engagement, its construct validity, and its relationship to academic performance.

## Study 1

Beginning as a design-based study for 3^rd^ grade, the ML-PBL intervention underwent several rounds of revisions and testing over the course of 4 years, including teacher experiments, classroom pilots, a field-test, and most recently an efficacy study to determine whether the ML-PBL intervention enhanced students’ science academic, social, and emotional learning. A randomized control trial was conducted in 46 Michigan schools (23 treatment and 23 control) which included four regions in the state. The final analytic sampled included a total of 2,371 students. The treatment condition included curriculum materials and professional learning experiences for teachers. To assess if there was a significant difference in academic science learning, a three-level hierarchical linear model (HLM) was conducted. This method was used to account for nesting of students within classrooms within schools. Results showed that the treatment students outperformed the control students by a .277 standard deviation on an objective summative test which is a substantial treatment effect (Krajcik et al., in press). This could be interpreted as a ten-point increase on a hundred-point scale or based upon a chosen percentile ranking in which the treatment could move the student from below proficient to proficient (Kraft, 2020).

The above work also investigated specific research questions related to social and emotional learning, specifically, whether the treatment support more positive responses on measures in self-reflection, collaboration, and responsibility for their own and others’ work. It is important to underscore that few studies measure elementary school students social and emotional learning in their science classes (National Research Council, 1999). Given these constraints, the team consulted relevant limited science studies of young children and more broadly: psychological research studies on SEL; developmentally appropriate questions for 3^rd^ graders; and items from other national assessments (e.g., the Early Childhood Longitudinal Study [ECLS-K] 2016; Durlak et al., 2015; Baines et al., 2017; Jagers et al., 2018). Recognizing differences in literacy skills among students, a drawn thumbs-up (agree), thumbs-down (disagree), and closed fist (neutral) were used to measure agreement. Students circled their feelings on a paper/pencil form administered in spring semester. Prior to the efficacy study, the SEL instrument was designed, field-tested, and revised. Confirmatory factor analyses (CFA) were performed and supported three key latent constructs: self-reflection, ownership, and collaboration (see Krajcik et al., 2021). Additionally, the reliabilities of these constructs were estimated to be 0.78 for self-reflection, 0.81 for ownership, and 0.74 for collaboration. Results from the efficacy study of the ML-PBL intervention showed that the treatment students were estimated to have 0.544 higher factor scores in reflection, 0.434 higher factor scores in ownership, and 0.416 higher factor scores in collaboration than the control students (Krajcik et al., 2021). These results indicate that it is possible to obtain validated measures of young children’s SEL responses for selected constructs. And in this instance, constructs that are specifically designed to be contextually relevant for particular SELs that the intervention was expected to impact.

As mentioned above, few studies have been able to examine the impact of engagement on elementary science learning. We chose to further examine the relationship between engagement and achievement as research has shown positive relationships between students’ determination to be engaged in the classroom and science achievement (Grabau and Ma, 2017). How students are feeling at the time of the lesson or activity can play a major role in how well they learn or understand key concepts. To explore student responses to project-based and three-dimensional learning, we developed optimal learning surveys that allowed us to measure student engagement in a repeated measures design. These surveys obtained student responses ‘*in situ*’ within the science classroom, capturing students’ perspectives on specific lessons as they happen (Csikszentmihalyi and Larson, 2014). Results of this new development study are described below.

During the beginning and first year of the Covid-19 pandemic, the ML-PBL team were able to observe students in their science classes *via* video, in-person, or a mixture of the two to collect data on the students’ engagement and teacher implementation. One of major observations from the videos was the variation of students’ engagement in their science lessons. Having identified in earlier studies of secondary students a set of constructs (i.e., interest, skill, and challenge) that showed increases in engagement and impacted science learning (see Schneider et al., 2016, 2020), the research team decided to pilot whether these same engagement constructs could be found in elementary science classes and whether they might also positively influence students’ science learning.

### Research questions

The research questions for this new development study include:

1. Can 3^rd^ graders reliably produce measures of interest, skill, and challenge ‘*in situ*’?
2. When studied with repeated measures, do interest, skill, and challenge load onto a single construct of engagement?

### Method

Using the same constructs of interest, skill, and challenge as fundamental dimensions of engagement, during the pandemic, the team developed a new methodology and series of items for 3^rd^ graders that relied on data collected situated in specific lessons within each unit. Keeping with the idea of measuring social and emotional learning ‘*in situ*,’ specific items were contextualized to be consistent with the lesson learning goals and how teachers may have been adapting them in the four units (see Bartz et al., 2022).

### Instruments/measures

For each unit during three different time periods, students were asked questions pertaining to specific measures of interest, skill, and challenge (see page 7 for fuller description). The three different time points were chosen based on the goals of each lesson, allowing us to collect more data from lessons that focused specifically on driving questions, investigation, building a model, or creating a final artifact. These items are situated directly in the context of each lesson. Six focal lessons, which contained the following features: driving question, modeling, investigation, and development of a final artifact, were sampled. For example, in the beginning of the toy unit after observing a toy rocket and how it moves, students were asked for interest, “I like asking questions about how the air rocket moves;” for skills, “I can ask questions about how toy rockets move the way they do;” and for challenge, “I had to think a lot to ask new questions about how rockets move.” With respect to collaboration, the students were asked, “When I worked with my classmates, we came up with different questions about the way the toy rocket moved;” and for ownership, “The questions I asked about the air rocket’s motion were important to me and my classmates.”

The data collection procedures used for measuring this engagement measure followed the original collection of the SEL survey, but with greater frequency. Teacher administered the four-question OLM survey to third grade students immediately following the lesson. The first three questions were based on engagement: interest, skill, and challenge. The fourth varied by form (A, B, or C) and rotated between collaboration, persistence, agency, time and outcome by lesson. A 4-point Likert scale was used (strongly disagree, disagree, agree, strongly agree) with students circling icons of thumbs up and thumbs down. In the pilot of the SEL measures at the elementary school level, at three different times during each of the four units, the teachers hand out paper copies of the engagement questions to the students in their class. The teachers then read aloud each of the questions, one at a time. After each question is read, students circle the corresponding thumb icon on their paper. In the cases where students circled more than one response, in the median score of responses was recorded.

### Sample

The sample for this analysis came from 25 3^rd^ grade classrooms in Michigan and included 596 students with a total of 3,369 responses for an average of 6 repeated measures per student.

### Analysis

Their responses to the engagement questions across the four ML-PBL units were analyzed. For the reliability of this survey, a Cronbach’s alpha was used to estimate the reliability.


$$\mathrm{alpha}=\left(\frac{K}{\left(K+1\right)}\right)\left(1-\frac{\sum Vi}{Vt}\right).$$

For understanding whether the interest, skill, and challenge loaded onto a construct of engagement, a confirmatory factor analysis was conducted. Factor loadings for each item onto this construct were estimated.

### Results

The descriptive statistics from the survey, including the items of interest, skill, challenge, and an additional question, are reported in Table 1.

**Table 1:** Sample descriptives.

	N	Mean	St. Dev	Min	Max
Interest	3,369	3.29	0.87	0	4
Skill	3,330	3.25	0.84	0	4
Challenge	3,367	2.79	1.08	0	4
Q4	3,362	3.18	1.01	0	4

A confirmatory factor analysis confirmed a unidimensional model with the following factor loadings for: interest (0.77); skill (0.41); and challenge (0.26). The overall reliability of the engagement measure is a Cronbach’s Alpha of 0.53. The overall reliability and item level reliabilities are reported in Table 2.

**Table 2:** Reliability using Cronbach’s Alpha.

	Item-test	Item-rest	Avg. interitem cov	Alpha
Interest	0.67	0.39	0.17	0.4
Skill	0.62	0.34	0.2	0.44
Challenge	0.61	0.21	0.25	0.56
Q4	0.69	0.35	0.17	0.42
Test scale			0.2	0.53

Additional analyses are being undertaken to study variation in engagement by lesson activities and individual level variables.

## Study 2

The secondary school intervention, “Crafting Engaging Science Environments,” (CESE) is a high school chemistry and physics PBL intervention similar to but independent of the elementary intervention. Both interventions meet the NGSS performance expectations and incorporate NRC three-dimensional learning and principles of PBL. CESE was administered to a diverse group of over 4,238 students in chemistry and physics classes in 70 high schools. The design like the elementary study was an efficacy study that involved a randomized control trial in California and Michigan. This intervention also included curriculum materials and professional learning for the teachers. Results were estimated using a two-level HLM with the outcome being the student level performance on the physical science items from the Michigan State Science Assessment and the main predictor of interest being treatment at the school level. For this estimation, a pretest and student demographics were included as covariates. Results show that treatment students, on average, performed 0.20 standard deviations higher than control students on an independently developed summative science assessment (Schneider et al., 2022). These results, like the ML-PBL, are quite large especially considering the advanced subject matter of the units and that they only extended over a 12-to-16-week period. Mediation analyses show an indirect path between teacher- and student-reported participation in modeling practices and science achievement. Exploratory analyses, using a two-level mixed logit model also indicate positive treatment effects for enhancing college ambitions. Overall, results show that improving secondary school science learning is achievable with a coherent system comprising teacher and student learning experiences, professional learning, and formative unit assessments that support students in “doing” science.

A major part of the study was investigating why secondary students, as shown in national and international studies fail to be engaged in their science classes which likely affects their interest in science learning, achievement, and science career ambitions (National Assessment of Educational Progress, 2021; Organisation for Economic Co-operation and Development, 2020). This question of how to enhance engagement in science was a major concern of the secondary school science study. Several major hypotheses about studying engagement were assumed at the onset of the study as discussed above that students’ social and emotional experiences at school are fluid throughout their daily lives (Csikszentmihalyi and Schneider, 2000). First, as discussed above students’ social and emotional experiences at school are fluid throughout their daily lives. It is not expected that students would be fully engaged in all their classes full-time any more than it is expected that adults would be consistently fully engaged in all activities at work or at home. Moreover, because of what is known about adolescent development, trying to create activities that keep teenagers fully engaged requires quite a high bar of motivation (Immordino-Yang, 2015). Irrespective of the barriers and challenges, the problem to be addressed in this study was creating environments that were engaging. The nature of science requires inquiry-based discovery (National Research Council, 2012; National Academis of Sciences, 2018); therefore, students may be more receptive to doing science than memorizing facts or plugging in equations.

The PBL framework, which stresses solving personally meaningful questions and encouraging instructional activities that require collaboration and are intellectually challenging, was ideally suited to test the constructs of engagement and their impact on academic science achievement. The work is situated in the work of Fredricks and McClolskey (2012) that identifies engagement as having cognitive, behavioral, and subjective components. Extending their definition, the new conception of engagement begins by identifying special behavioral activities that are temporal in quality, spark personalized interest, require competence of a set of knowledge and experiential science practices, and undertake challenging problems.

In contrast to those who have conceptualize engagement as a general trend, this model of engagement identifies engagement as domain specific in duration and in intensity, which fits more closely with current definitions of situational interest in science learning (see Lavonen et al., 2005; Krapp and Prenzel, 2011). This situational approach is different from other scholars who are interested in identifying universal traits (Deaux and La France, 1998; Cuddy et al., 2008). These engagement experiences are defined as optimal learning moments, which also builds upon the idea of “flow” defined by Csikszentmihalyi and Csikszentmihalyi (1988) as situation specific instances when an individual is so deeply involved in a specific task-related activity that time flies by (Csikszentmihalyi, 1990; Hektner et al., 2007).

The PBL curriculum, as discussed above, begins with a driving question when students are in specific situations and faced with a problem or phenomenon that is relevant and meaningful to their lives, such as: “how can I build a safer car?” To build that car, students need to have the necessary knowledge and skills to create a solution. Irrespective of the students’ skill level, finding a reasonable solution should be a challenge, one that sparks determination. When students are fully engaged in a learning task, this is defined as an optimal learning moment (OLM). These moments do not just happen, but need to be artfully constructed and coherent, which is yet another fundamental aspect of PBL which inspires the acquisition of new knowledge, the use of imagination, and stretching problem-solving abilities.

Optimal learning moments can be verified and understood by other related subjective experiences occurring at nearly the same time. For example, it is expected that when involved in these activities’ students feel successful, confident, active, happy, and enjoyment with the activity (Shernoff et al., 2003; Shumow and Schmidt, 2014). Learning accelerants are those experiences of feeling anxious or stressed, which activate learning (Csikszentmihalyi and Schneider, 2000). Finally, the contrast to positive subjective experiences, termed learning detractors, is when students involved in an activity feel confused or bored and are therefore less likely to be actively engaged or experience an OLM (Csikszentmihalyi, 1990; Schneider et al., 2016).

During the field test of the CESE intervention, an ‘*in situ*’ study of social and emotional relationships to science achievement was conducted with the ESM. The data included 8,273 responses from 244 students in 15 classes taught by 14 teachers in Michigan. Only half of the variance in determination and giving up were at the student level, meaning that both feelings are not altogether stable student traits and most importantly environment and context matters (Schneider et al., 2020). Students were more likely to report giving up when tasks became more challenging, but at the same time, when classroom activities were reported as more challenging than average, students were more likely to persevere, suggesting that determination is partially situationally dependent and shaped by what is occurring in the types of activities presently involved in either with others or oneself.

While these ESM results were promising, there were several limitations. This was a pilot not a randomized trial where students in a treatment and control group could be compared. Rather it was the case that measures of engagement and feelings regarding challenge were measured using a single case design, where each classroom acts as its own experimental control (vacillating from treatment periods to times in the classroom when it was “business as usual”). These repeated periods were assessed to determine if the treatment influenced students’ engagement. Although, the pilot study results showed that more engaged students had higher grades it could not be directly attributable the CESE intervention. However, the positive nature of the results prompted the team to use the ESM in the future efficacy study (2018–2019) in selected treatment and control classrooms (Schneider et al., 2020).

Preliminary results on the measures of engagement show that when considering levels of interest skill, and challenge, student engagement levels increase and are accompanied by other positive social and emotional affects, as well as decline in feeling of boredom and confusion. These findings show that concepts such as engagement, creativity, and problem-solving are situationally specific and share nearly equal variance when contrasted with person-level characteristics. In other words, even if a student is not interested in a topic or whose previous science achievement scores are below average, a carefully created situation can alter their negative predilections toward science, bringing considerable strength to the “nurture” side of learning especially when breaking from traditional types of assessment memorization and instead using imagination, problem-solving, and taking different points of view into consideration when engaged in scientific practices. However, these are preliminary results and an important question is the level of challenge and what impact it has on motivating higher engagement and learning for all students in specific contexts. (see, Schneider et al., 2020, for a deeper discussion of these ideas and how they were conceived and measured in the earlier field study).

### Current study

Most recently, a deeper examination of “challenge experiences” in science class has been conducted (Bradford and Bartz, 2022; Chen et al., 2022). Until now, challenge has not been a major state in the psychological literature, and less attention has been placed on perceived “challenge” experiences in science classroom environments. Challenge experience can be highly motivating and encourage deeper engagement in a classroom task. However, there is less research regarding the importance of perceived challenge for high schoolers, how it varies ‘*in situ*’, and how students react to challenging experiences. To fill the gap, this study has two different analyses.

The first starts with a *case study approach* to illustrate a particular pattern of perceived challenge by visualizing three student cases in 4 days of their school life. The visualization focuses on a precise moment in time and provides corresponding details on where students were, what they were doing, and who they were with. From there, the graphic visualization considers students’ school life for 4 days and how this pattern of perceived challenge experiences is general or unique to individuals who vary in their background science knowledge. After visualizing three students’ life in school, we use another graphic layout to visualize students’ reactions to perceived challenges in their positive and negative states. The purpose of this visualization work is to lead the researchers to discover patterns of emotionality shared by several members of the student sample for 4 days.

The second analysis uses data from a sample of students from the field test and efficacy study to understand the use of ESM and student’s variation in emotions across years. These analyses employ a series of repeated measures estimation of students situational perceived challenge, stress, anxiety, determination, giving up, and confusion to understand how the relationship between challenge and giving up and confusion is mitigated by stress and anxiety. We assume that challenge is important in driving learning; however, if challenge is correlated to giving up and confusion, this would lead to a negative relationship between challenge and learning. This leads to a question of whether anxiety and stress may be stronger mediating factors in the relationship between challenge and giving up and confusion.

#### Research questions

The research questions for study 2 were:

1. How does perceived challenge vary by individual students?
2. How does the relationship between perceived challenge and positive and negative emotions vary by individual students?
3. What is the relationship between students’ perceived challenge coupled with stress and anxiety and determination, giving up, and confusion?

#### Sample

During the field test of the CESE (2013–2018), a total of 867 students were reported with the ESM. For the efficacy study (2018–19), a total 545 students were reported with the ESM for a total of 1,412 students combined. The phones were programmed to alert the students randomly 6–8 times per day (at least 3–4 times when they had science lessons) over an assigned period. An initial ESM prompt would occur in the beginning, mid- and late point of a study session automatically set up by researchers using the PACO app. Students were asked to respond to an identical questionnaire (nearly 30 items) within a 15 min window. Two reminders would occur 10 and 15 min after the initial prompt. On average, it takes about 90 s to complete items. Each day all participants received eight to 10 beeps on their smartphones which gave them 40 total response opportunities during a study period. We preprogrammed the beep schedule randomly and guarantee a minimum of 1–3 beeps occurring in science classes, resulting in 5 to 15 beeps per person in this study. In total, the data comprised 3,234 responses. The average valid beeps per student is 6 in science classes. We conducted two separate analyses, one which only analyzed the students in the efficacy study and a second analysis from both the field and efficacy studies.

The first analyses reported is from the efficacy study which contained a diverse population of students living in both Michigan and California with an overrepresentation of students for whom English is not their first language, as one of our sites was a mile from the Mexican border. Among the efficacy students’ sample, 315 (58%) had valid student background information, including Race/Ethnicity, gender, challenge experiences and science pretest scores. This student background survey was collected at the beginning of the year *via* a Qualtrics Survey. Table 3 reports the descriptive statistics for the students. Of the 315 students who provided valid ESM responses, the racial composition of the groups was 60% White, 8% Black, 17% Hispanic, 5% Asian and 5% multi-racial.

**Table 3:** Descriptive statistics of student sample.

	Freq.	%
Male	151	48.55
Female	160	51.45
Grade 10	81	31.89
Grade 11	146	57.48
Grade 12	27	10.63
White, (non-hispanic)	200	60.4
Hispanic	57	17.2
Black	28	8.5
Asian	18	5.4
Other	11	3.3
Multiracial	17	5.1
Total valid student info	331	
Demographic info Missing	214	
	Mean	SD
Percentile ranking of pre-test	62.69	22.03
Challenge	2.31	0.74

The second analysis used the entirety of the sample from both the field and efficacy tests (demographic information is unavailable for this combined sample; however, the sampling scheme for the field and efficacy tests targeted schools with significant numbers of low-income and minority students). The entire sample was used in the second analysis which uses aggregate statistical modeling to understand the validity of these relationships across many years (2013–2019).

### Methods

#### Instruments and measures

To measure engagement, studies typically employ surveys which are rarely conducted ‘*in situ*’ or when they are happening, which of course fails to capture how students are feeling from one moment to the next. Measuring how students feel across moments allows us to identify when they feel successful at what they are doing and its relationship to what they are learning. The ESM records what students are doing, what they are thinking about, and what they report feeling in the moment forming an archival repository of daily experiences. This focus on the situational and contextual aspects of what happens in-and -out-of-the classroom lessens the opportunity for recall bias and socially desirable answers and has been validated in previous studies (Hektner et al., 2007). The ESM SEL survey items and their response are reported in Table 4. There were approximately 30 items.

**Table 4:** CESE ESM instrument.

**ESM questions in CESE**
Q1 Where were you when you were signaled?
Q2 What science class were you in?
Q3 Which best describes what you were doing in science when signaled?
Q4 What were you doing when signaled?
Q5 What were you learning about in science when signaled?
Q6 Who were you with?
Q7 Were you doing the main activity because you…
Q8 Was what you were doing…
Q9 Were you interested in what you were doing?
Q10 Did you feel skilled at what you were doing?
Q11 Did you feel challenged by what you were doing?
Q12 Did you feel like giving up?
Q13 How much were you concentrating?
Q14 Do you enjoy what you are doing?
Q15 Did you feel like you were in control of what you were doing?
Q16 Were you succeeding?
Q17 Was this activity important for you?
Q18 How important is this activity in relation to your future goals/plans?
Q19 Were you living up to the expectations of others?
Q20 Were you living up to your expectations?
Q21 I was so absorbed in what I was doing that time flew.
Q22 How determined were you to accomplish the task?
Q23 Were you feeling…Happy
Q24 Were you feeling… Excited
Q25 Were you feeling… Anxious
Q26 Were you feeling… Competitive
Q27 Were you feeling… Lonely
Q28 Were you feeling… Stressed
Q29 Were you feeling… Proud
Q30 Were you feeling… Cooperative
Q31 Were you feeling… Bored
Q32 Were you feeling… Self-confident
Q33 Were you feeling… Confused
Q34 Were you feeling… Active

In both analyses, students were beeped several times a day (7 times) during a week both inside and out of school and classes, with several more signals in science classes. Each classroom was randomly chosen for a specific week(s) during the intervention for data collection. Each data entry has a time stamp to indicate when the responses was collected. This approach is different from single survey as it records a set of repeated specific social and emotional measures interacting with specific activities, such as, doing a hands-on experiment in science class as compared to playing a video game. These responses are uploaded to a secured server which sends information to a cloud and are then quickly transformed into clean datasets and ready for analysis. Confidentiality is maintained by student anonymized identification numbers (It is important to note that all of our data collection and analyses underwent Institutional review board approval and received exempt status).

**Figure 1:**
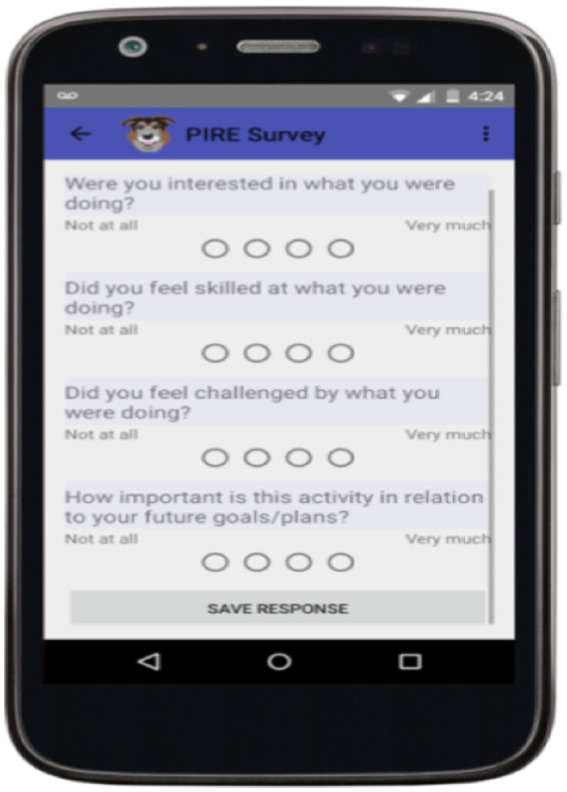
Screen shot of PACO app (Evans, 2016). Republished with permission.

Figure 1 below shows a screen shot of one of the questions used in the secondary school intervention in both the field test and efficacy study. The actual software program was developed by Robert Evans, a google engineer, who named the program Paco,^1^ after his dog which barks to let students know it is time to answer the questions. Although there are multiple questions, the students can move through them quickly. Since they are programmed for smartphones, beeping schedules can be easily programmed over the course of days of a week or multiple weeks during specific time periods. Figure 1 shows the questions asked regarding interest, skill, and challenge.

#### Analysis

**Figure 2:**
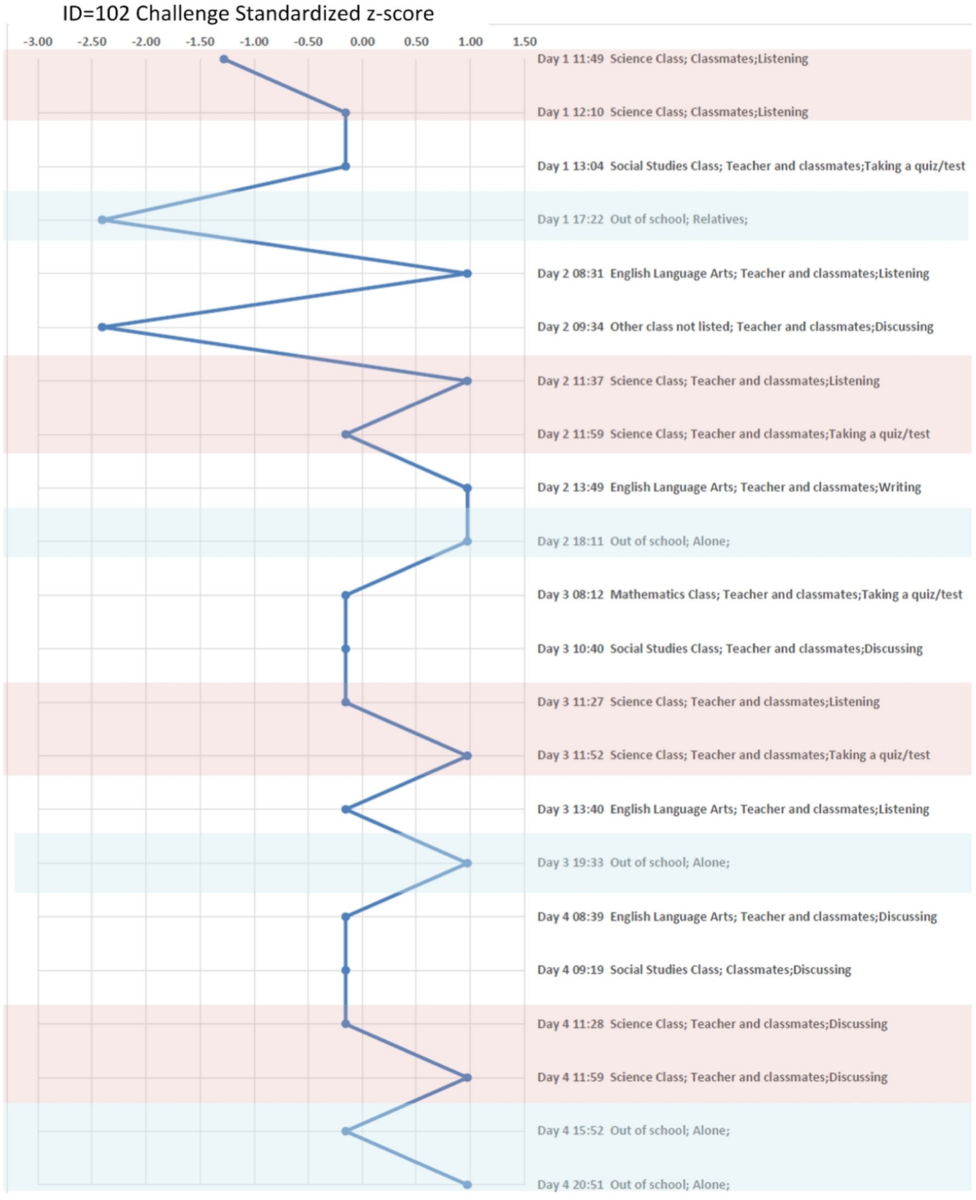
Dennis (Low-performing student) Experienced “Challenge” in situ across Context. Report Z-score Over 4 days. Pink color marks the moments in science classroom, and the light blue color marks the moments when a student is out of the school.

**Figure 3:**
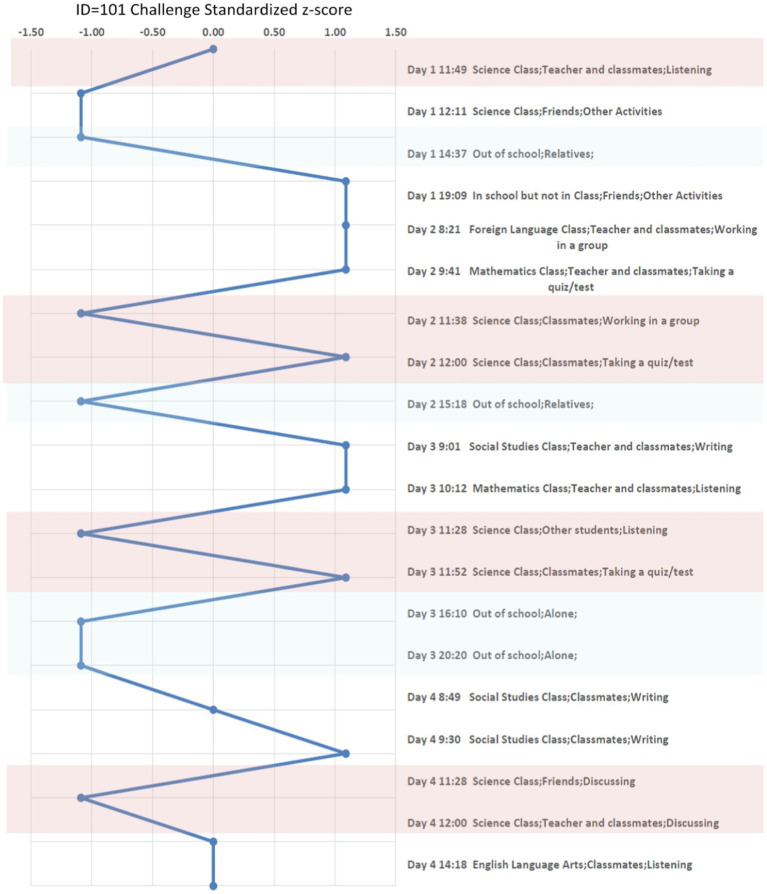
Megan (Average student) Experienced “Challenge” in situ across Context. Report Z-score Over 4 days. Pink color marks the moments in science classroom, and the light blue color marks the moments when a student is out of the school.

**Figure 4:**
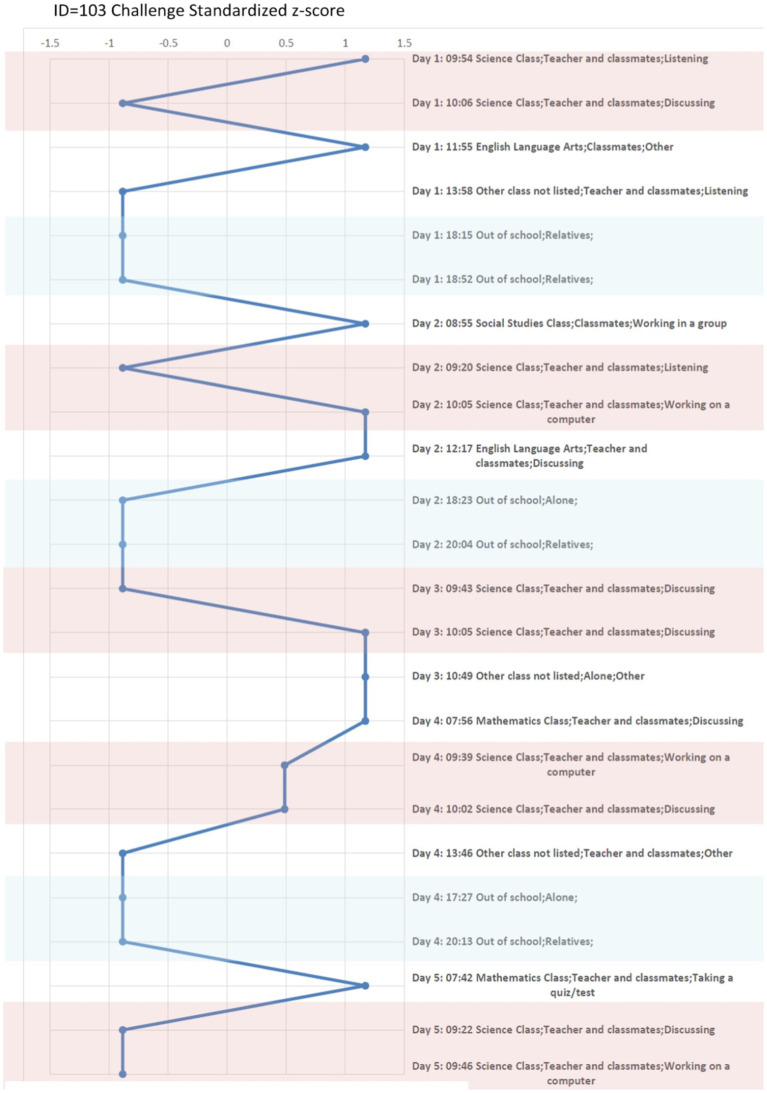
Collins (Above Average student) Experienced “Challenge” in situ across Context. Report Z-score Over 4 days. Pink color marks the moments in science classroom, and the light blue color marks the moments when a student is out of the school.

A more in-depth examination of ESM is shown in the case study analysis. A second analysis which relies on a hierarchical linear model (HLM) was used for the aggregate study of the field and efficacy tests. Beginning with the in-depth case study, three students were selected among varying levels of school achievement to analyze their variability in emotionality with graphic visualization. First a description is given for how an individual student experienced challenges across activities, locations, and companionship in their 4 days. Second, the three students’ emotional responses within each person’s positive and negative states when challenged is shown in Figures 2–4. Finally in the second analysis, we explore the relationship of challenge on spurring continued determination or on confusion and giving up with or without changes in other states of emotionality through the HLM.

#### Three case studies

To understand the situational and individual differences for the students in the case study, their ESM responses were obtained throughout the day, including an oversample of beeps in their science classes (Chen et al., 2022). Table 5 shows the three students’ background, the level of prior test scores, and the average perceived challenge across all contexts and in the science classroom only. To see how this visualization works, consider the rating of perceived challenge by those three students. The three students are: Dennis, a low-academic performing student based on his prior test scores; Megan, an academically average student; and Collins, an above average on his prior test scores. During the 4 days of the study, Dennis has an average challenge response of 3.14 across all contexts and the average challenge response of 3.25 in the science classroom. Megan has an average challenge response of 2.51 across all contexts and the average challenge response of 2.25 in her science classroom. Collins has an average challenge response of 2.29 across all contexts and the average challenge response of 2.30 in his science classroom. These three students are in the same science class at their school. Examining these individual case studies allows for the comparisons among the three students, their different social and emotional experiences throughout the day, and their relationship to challenge in different contexts.

**Table 5:** Three student case study.

	School performance	Gender	Average Perceived challenge (individual) all context	Average Perceived challenge (individual) in science classroom	n of moment
Dennis	Low-performing student	Male	3.14	3.25	22
Megan	Average performance	Female	2.51	2.25	20
Collins	Above average	Male	2.29	2.3	24

A standardized z-score (mean of 0 and standard deviation of 1) of perceived challenge was calculated and took into account individual differences while also allowing for comparison across individuals on a common scale. The z-scored perceived challenge also provides an advantage for exploring the emotional response in different contexts. The students’ z-scores of challenge are compared across different settings and activities in these case studies. Additionally, the students’ positive emotional states, which are measured by “happy, enjoy, excited, success and competitive,” and negative emotional states, which are measured by “angry, stress, confused, give-up and anxious,” are compared across different levels of the students’ challenge levels using a different visualization. An average score of five emotional responses was used to represent the positive and negative states for the three cases. The five positive and negative emotional states were chosen based on earlier work (Hektner et al., 2007, pp: 110–123). These analyses are depicted through graphs to illustrate these varying states of challenge with the students’ other positive and negative emotional states. These analyses give insight at the individual level; however, to understand aggregate relationships, we move to statistical models with the entire sample of ESM students from the field test and efficacy study.

#### From case studies to a statistical model

The ESM asks students questions that correlate perceived challenge experiences that may confound the relationship with other positive and negative psychological states. Therefore, it is important to understand the influence confounding variables may have on students perceived social and emotional well-being. More specifically, in the case of perceived challenge, this new work has begun to examine the confounding effects of stress and anxiety on the relationship between challenge and two important negative psychological states, confusion and giving up, and one positive state of determination (Bradford and Bartz, 2022). First, the correlations for the variables were calculated to understand the relationship between challenge, stress, anxiety, confusion, giving up and determination.

Then, using a repeated measures HLM, the relationship between challenge and confusion, giving up, and determination was explored first without covariates and then with stress and anxiety as covariates. The following two equations were estimated.

Model 1:


$$\mathrm{outcom}e_{ij}=\delta_{00}+\delta_{10}\mathrm{challenge}+\nu_{0j}+\epsilon_{ij}$$

Model 2:


$$\begin{array}{c} \mathrm{outcom}e_{ij}=\delta_{00}+\delta_{10}\mathrm{challenge}+\delta_{20}\mathrm{stress}\\ \;+\delta_{30}\mathrm{anxiety}+\nu_{0j}+\epsilon_{ij} \end{array}$$

Where δ10 is the relationship between challenge and the outcome in the two models, v_0j is the student level random intercept and epsilon_ij is the beep level error term. The $\delta_{10}$ from both models 1 and 2 were compared using the Hausmann test to determine if the inclusion of stress and anxiety significantly changed the relationship between challenge and the outcome.

### Results

#### Three case studies

In Figure 2, we plot the z-score of Dennis’ perceived challenge over 22 moments. We also plot the z-score of Megan’ perceived challenge over 20 moments in Figure 3 and the z-score of Collins’ perceived challenge over 24 moments in Figure 4. The three plots center in the middle line of the z-score as 0, which is each individual student’s average challenge score. Dennis has a smaller range of perceived challenge than Megan and Collins, and he favors to report high perceived challenge among 4 days of the study. If we are interested in the context of science classroom, we can compare patterns when the three students are taking quiz in the same context. For example, we use the pink color to mark the moments in science classroom, and the light blue color mark the moments when a student is out of the school. Dennis and Megan perceived higher challenges, particularly when taking a quiz (z-score ranged from 0.5 to 1.0). Collins feels less challenged when taking a quiz but experiences a higher challenge in group discussion or when using a computer. We can conclude that Dennis and Megan objectively have higher perceived challenge than Collins when taking a quiz among the 4 days they were sampled.

Relative to the science classroom context, the out-of-school context (colored in light blue), Megan and Collins are less challenged especially when compared to Dennis. Overall, these three case studies show that the context of when students feel challenged can vary considerably by individuals and activities.

**Figure 5:**
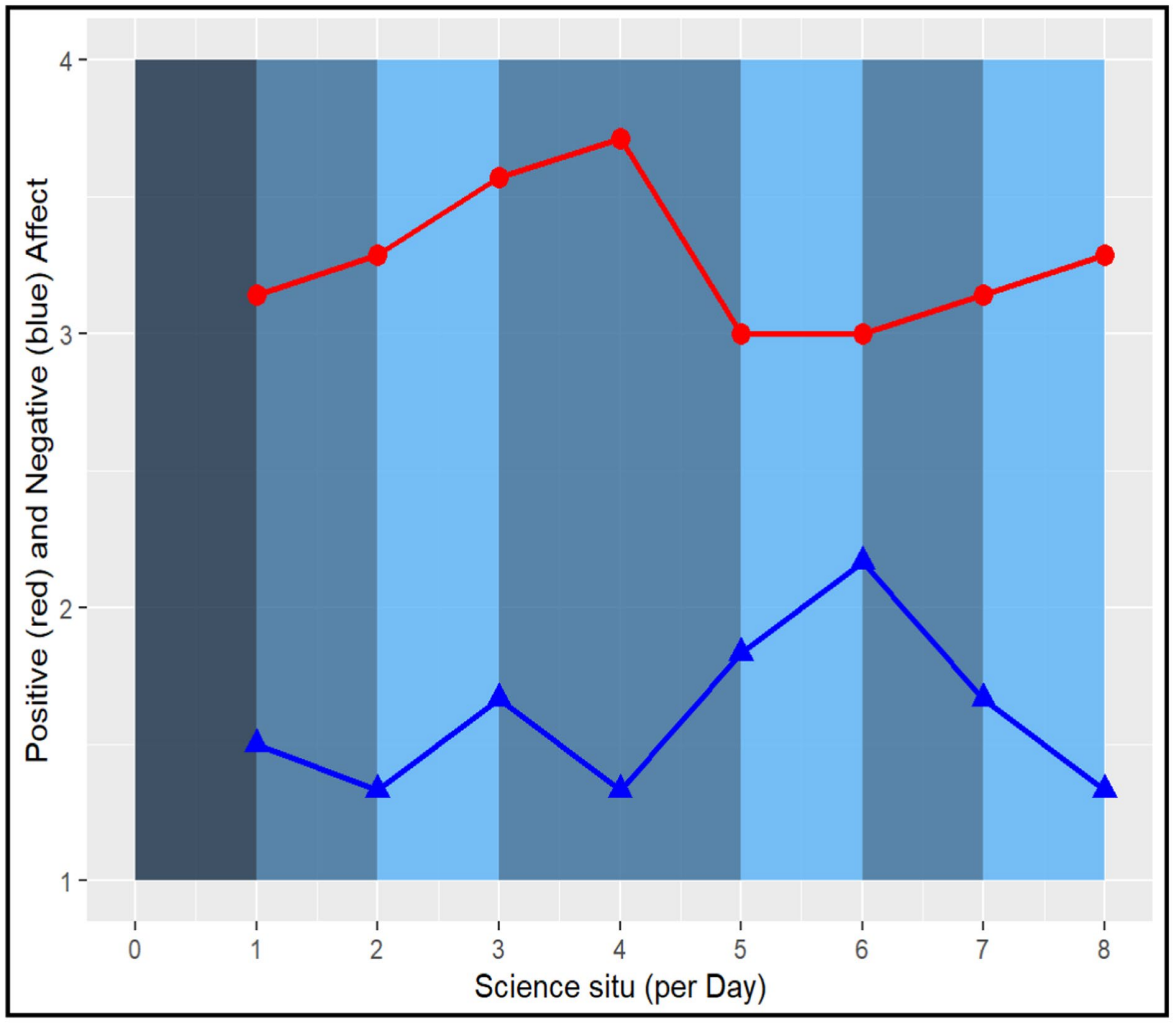
Dennis (Low-performing student) Experienced Challenge in Relation to Positive and Negative Psychological States. Report raw scores in positive and negative emotions. The dark blue color marks the lowest challenge moments (=1), whereas the light blue color marks the highest challenge moments (=4) in the science classroom.

**Figure 6:**
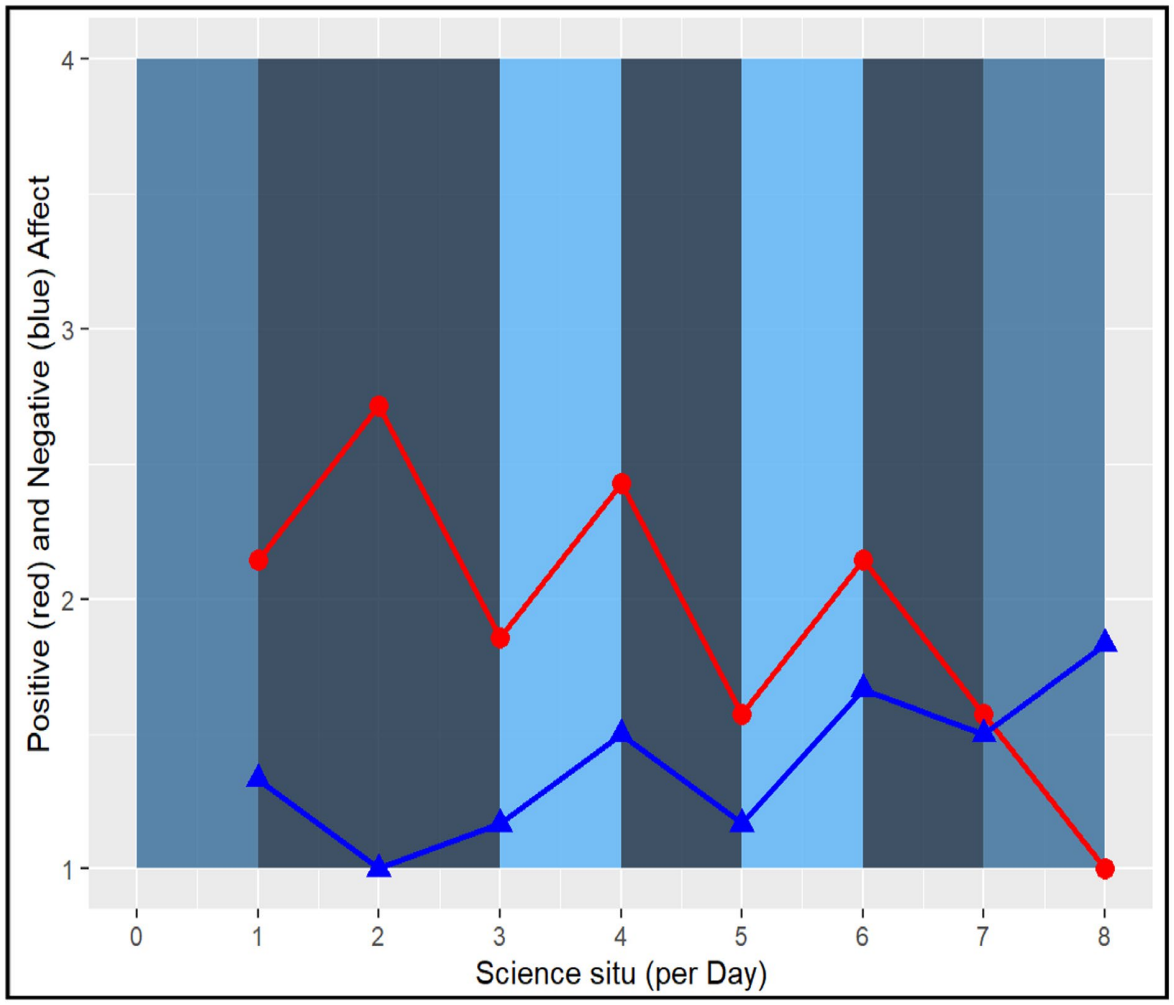
Megan (Average student) Experienced Challenge in Relation to Positive and Negative Psychological States. Report raw scores in positive and negative emotions. The dark blue color marks the lowest challenge moments (=1), whereas the light blue color marks the highest challenge moments (=4) in the science classroom.

**Figure 7:**
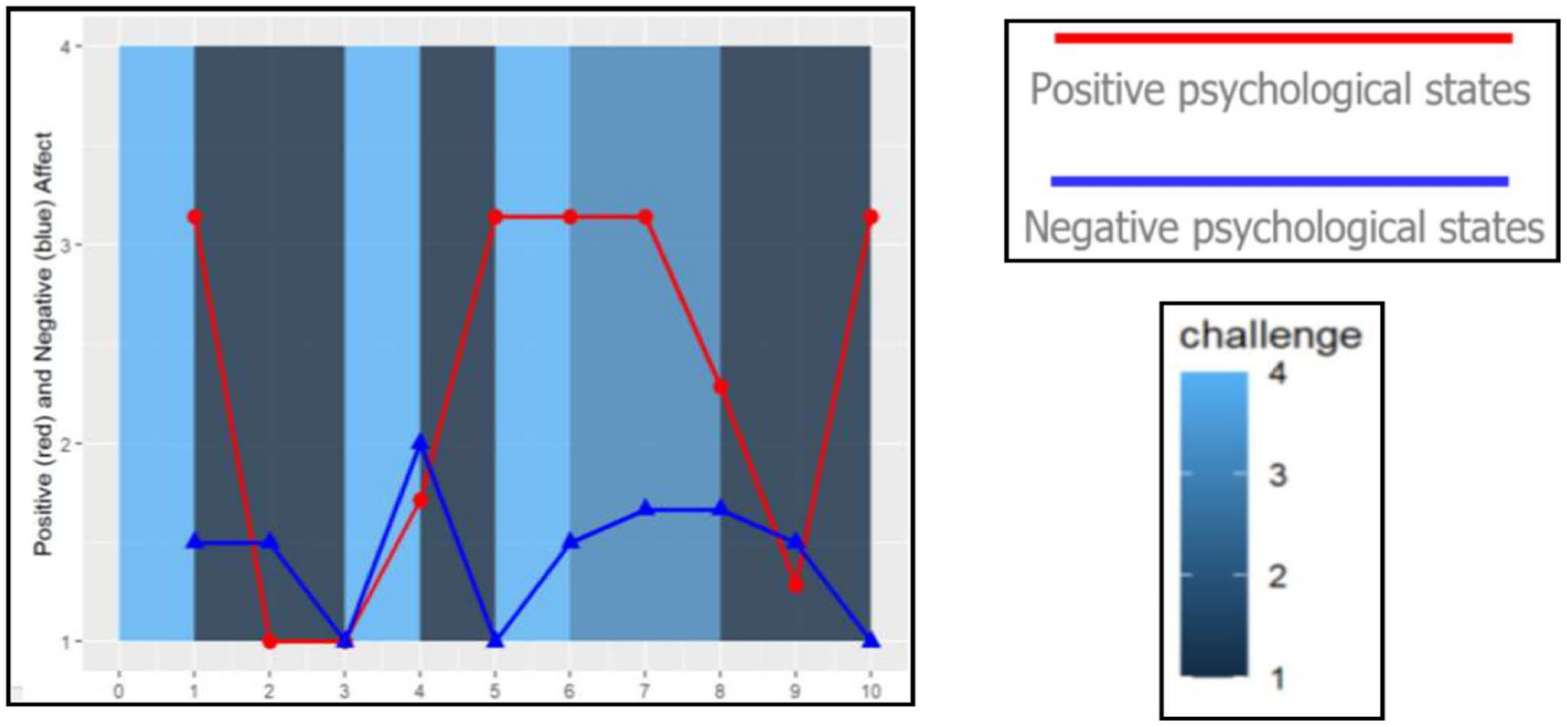
Collins (Above Average student) Experienced Challenge in Relation to Positive and Negative Psychological States (Low-performing student). Report raw scores in positive and negative emotions. The dark blue color marks the lowest challenge moments (=1), whereas the light blue color marks the highest challenge moments (=4) in the science classroom.

Recognizing the individual variability of experiencing challenge across contexts, the next question is whether emotional responses related to challenge differ by student. When challenged, is this experience more positive for Collins and Megan than Dennis, or do they all report similar feelings? Figures 5–7 show other positive and negative emotional states of these three students as well as their level of challenge during their science classes.

Among our three student cases, Dennis had fewer positive psychological states and reported more challenging tasks during his science class. His perception of high challenge (light blue bar) is less correlated with positive psychological states and more correlated with negative ones. Additionally, Dennis’ psychological states fluctuated more than the two other students. These fluctuations are more apparent when experiencing positive psychological states (e.g., feeling successful, confident) than his negative psychological states of confusion.

Megan, on the other hand, had a declining trend of positive psychological states over the 4 days. When she experienced high challenge tasks in the science classroom, her negative psychological states increased. However, for Collins, the above average student, his positive psychological states were more correlated with higher challenge. Additionally, during moments of rising challenge, Collins experienced other positive emotions. Overall, the relationship between challenge and positive and negative psychological states seem to vary across students and days (Chen et al., 2022).

#### Statistical results from the entire sample

Among all students in the ESM sample, challenge was closely related to negative emotions for some students, while for others, it was closely related to positive emotions, and for others, there was no relationship. However, these results do not indicate how stress and anxiety might be influencing the relationship of challenge with other positive and negative emotions. Instead of focusing on all positive and negative emotions, a few key variables were explored more deeply: confusion, giving up, and determination, which were all positively correlated with challenge as seen in Table 6, which includes the entire sample of students, 1,412 students from the field test and efficacy study.

**Table 6:** Pairwise correlations of challenge, stress, anxiety, giving up, determination, and confusion.

	Stress	Anxious	Challenge	Give up	Determined	Confused
Stress	1.000					
Anxious	0.460	1.000				
Challenge	0.310	0.270	1.000			
Give up	0.430	0.310	0.380	1.000		
Determined	−0.028	0.093	0.220	−0.099	1.000	
Confused	0.54	0.400	0.420	0.490	−0.022	1.000

However, importantly, confusion and giving up were also positively related to stress and anxiety, while determination was not. Therefore, the question arose was whether stress and anxiety may be accounting for this positive relationship between challenge and confusion and challenge and giving up.

From the repeated measures HLM, the positive relationship between challenge and confusion and challenge and giving up significantly decreased in absolute value, when including stress and anxiety as covariates ( δ≈0.1,p−value<0.001 ), while the relationship between challenge and determination remained relatively the same (Bradford and Bartz, 2022).

## Discussion

The present findings extend previous research in at least two ways: First, these results provide a moment-level look at context differences in response to daily challenges in school, incorporating both the intra-individual as well as the inter-personal level across study one and study two. Both offer insights for each other to complete the puzzle of challenging experiences in students’ daily lives in school. These finding of significant context differences in intra-individual variability of experiencing challenge and other positive and negative states, to some degree, suggest that the relationship between perceived challenge, optimal learning moment, and psychological reactions is complex. Examining these relationships among these different emotions also offers that classroom learning, as we might have expected, is not a simple correlation with a specific experience but needs to be seen in context, over time, and in relationship to other events.

To further consider individual and contextual factors simultaneously, a designed statistical model like Simultaneous Equation Modeling (SEM) or Dynamic Structural Equation Modeling (DSEM) is essential to move this line of research forward. Second, complementing previous optimal learning moment literature on the states of the flow (Csikszentmihalyi and Csikszentmihalyi, 1988; Schneider et al., 2016), students’ determination could be one psychological state that may keep students working on the challenging tasks in science. The results from adding stress and anxiety as covariates indicates that as one explores individual and contextual differences in their experiences, one should also consider the confounding effects that may occur when these individuals are experiencing many different emotional states at once. Additionally, these results may suggest that stress and anxiety may not be as important of an activation for challenge. These results offer some possibilities for discovering methods to increase students’ optimal learning moments in science.

### Limitations of the study

With respect to specific limitations of study 1, there are few ‘*in situ*’ surveys for elementary level students for which we could compare our results. We have plans to use collected videos of classrooms to collaborate our findings which could increase the validity of these instruments. With respect to study 2, there may be other emerging technologies that could capture more changes in emotionality than the ESM, such as combining individual responses with video technology to capture facial, cognitive, and biomarkers, to which we could compare our results.

Overall, more studies are needed to use these techniques to build a corpus of work so that a comparison across studies can be examined to understand the reliability and validity of these techniques and their results. Despite our limitations of not having more in-depth analyses of personal and environment influences on social and emotional learning, our work provides another lens for understanding how levels of engagement and motivation are related to achievement, especially today when COVID’s effects on these important relationships need further exploration. Additionally, more studies are needed on emotionality in classrooms that take into account student cultures, family histories, race/ethnicity, and gender.

### Implications for raising interest in STEM

Even though classrooms are busy fluid learning environments, results show it is possible to measure social and emotional experiences, but they vary considerably by context. Some students find certain types of activities more interesting than others, and their skill levels vary meeting similar challenging problems with a diverse set of reactions from boredom and confusion to determination and sense of success and accomplishment. These variations by context indicate that isolating a specific measure of emotionality may overlook the factors at work that could be deterrents to motivation and persistence in STEM. It seems critical that researchers attempting to increase motivation for students to become engaged in learning experiences need to focus on the environment and emotions which operate at the same moments within the same context. And most importantly when considering engagement, recognizing that students vary in their skill levels, and this may be affecting the pursuit of learning new skills and attempting challenging STEM problems.

The greatest challenge for researchers who wish to transform STEM learning environments is determining the important types of social and emotionality constructs that make the most sense given the subject matter and experiences when students are expected to be engaged. This work has deliberately focused on science classrooms, where the underlying instructional and curricular activities are crafted in accordance with recent reports for transformative pedagogical practices. The toolkit of social and emotional measures being considered are those that seem the most reasonable given the goals of the lessons and the phenomena and problems to be solved. However, in trying to disentangle the behavioral, cognitive, and emotionality of engagement, considering interest, skill, and challenge are imperative, as well as other social and emotional factors that also occur when valuing teamwork and collaboration for having students learn and work with others and reach a place of ownership of ideas and products.

During the in-depth study of engagement (i.e., interest, skill, and challenge), several new factors related to learning have occurred. Interest, as others have also recognized, is critical; however, it must be constructed around ideas that the students find purposeful to their own lives. Memorizing the elements of the periodic table without knowing the purpose behind understanding the properties of the atom is a non- starter to a student. However, why we need to understand the relationship of certain elements to each other and their impact on chemical reactions experienced in everyday life can become more meaningful to a student.

Students have different skill levels and when choosing group experiences being attentive to the likely variation in the classroom is indispensable. The importance of bringing everyone into the problem-solving activity and making it a reasonable challenge for all students is likely to affect their personal as well as the groups’ continued work on a project or problem. The idea here is not to construct activities that have the lowest level of skills but rather to offer various flexible routes to problem solving for all the students. Nonetheless, it is the case in PBL that there are certain disciplinary core ideas that are regarded as critical and that has to the starting point of the lessons. What students need is an awareness of their own confidence to face a challenge and how that can fit into the space of figuring out a phenomenon or solving a problem.

Moving students to learn something they do not know changes the nature of learning from memorization to using ideas. This type of learning poses another set of ideas, in that students are taking on something that they do not know but they could find out. This process exposes their vulnerabilities in of not knowing—for which they need to learn to be more comfortable with. This is particularly problematic for females especially in adolescence, where taking risks and exposing one’s vulnerabilities is typically a positive aspect of the socialization process they encounter (Reniers et al., 2016). What is needed here is to underscore the value in taking intellectual risks in problem solving learning activities and the determination to continue working until a solution is found. Coming out of one’s comfort zone intellectually particularly in science where discovery and new innovations are fundamental must be nurtured not just with content but the social and emotional factors that can inspire motivated students to solve. Understanding these relationships among these different emotions suggest that classroom learning as we might have expected is not a simple one to one correlation with a specific experience but need to be seen in context, over time and in relationship to other events. This underscores the difficulty and limitations of new curricular packages designed to measure and relate emotionality to achievement and certain positive behavioral actions.

## Ethics statement

The studies involving human participants were reviewed and approved by Michigan State University IRB. Written informed consent from the participants’ legal guardian/next of kin was not required to participate in this study in accordance with the national legislation and the institutional requirements.

## Author contributions

BS: conception and design of work, drafting the article, review of data analysis and interpretation, and critical revision. I-CC: data analysis and interpretation, drafting the article, and critical revisions of the article. LB: data analysis and interpretation, drafting the article, and critical revisions of the article. KB: data analysis and interpretation, drafting the article, and critical revisions of the article. All authors contributed to the article and approved the submitted version.

## Funding

This study is supported by the National Science Foundation (OISE-1545684; PIs Barbara Schneider and Joseph Krajcik); George Lucas Educational Foundation—Lucas Education Research (PI Joseph Krajcik); and the John A. Hannah Chair in the College of Education at Michigan State University. Any opinions, findings, and conclusions or recommendations expressed in this material are those of the authors and do not necessarily reflect the views of the National Science Foundation and the George Lucas Educational Foundation.

## Conflict of interest

The authors declare that the research was conducted in the absence of any commercial or financial relationships that could be construed as a potential conflict of interest.

## Publisher’s note

All claims expressed in this article are solely those of the authors and do not necessarily represent those of their affiliated organizations, or those of the publisher, the editors and the reviewers. Any product that may be evaluated in this article, or claim that may be made by its manufacturer, is not guaranteed or endorsed by the publisher.

## Footnotes

^1^ https://www.pacoapp.com/

## References

[ref1] Baines A. DeBarger A. De Vivo K. Warner N. (2017). “Why is social and emotional learning essential to project-based learning?,” *LER position paper 2* United States: George Lucas Educational Foundation.

[ref2] Bartz K. Miller C. Bateman K. (2022). *Measuring Engagement in 3^rd^ Grade Science Classes.* Working Paper. Michigan: Create for STEM.

[ref3] Bradford L. Bartz K. (2022). Intersections of challenge, stress and anxiety: When is the challenge too much? [Conference Presentation]. AERA Annual Conference 2022, San Diego, CA, United States.

[ref4] Chen I. Bradford L. Bartz K. Krajcik J. (2022). Exploring the stability and fluctuation of experiencing “challenges” in the high school science classroom [Conference Presentation]. AERA Annual Conference 2022, San Diego, CA, United States.

[ref5] Csikszentmihalyi M. (1990). Flow: The Psychology of Optimal Experience. New York: Harper Perennial.

[ref6] Csikszentmihalyi M. Csikszentmihalyi I. (1988). Optimal Experience. New York: Cambridge University Press.

[ref7] Csikszentmihalyi M. Larson R. (2014). “Validity and reliability of the experience-sampling method” in Flow and the Foundations of Positive Psychology. ed. Peele-Eady (Dordrecht: Springer), 35–54.

[ref8] Csikszentmihalyi M. Schneider B. (2000). Becoming Adult: How Teenagers Prepare For the World of Work. New York: Basic Books.

[ref9] Cuddy A. J. C. Fiske S. T. Glick P. (2008). “Warmth and competence as universal dimensions of social perception: the stereotype content model and the BIAS map,” in Advances in experimental social psychology. ed. Zanna M. P., *Vol*. 40. (Oxford: Academic Press), 61–149.

[ref10] Deaux K. La France M. (1998). “Gender” in Handbook of Social Psychology. eds. Gilert D. T. Fiske S. T. Lindzey G., vol. *Vol*. 1. 4th *Edn.* (New York: McGraw-Hill), 788–827.

[ref11] Dorn E. Hancock B. Sarakatsannis Viruleg E. (2020). COVID-19 and student learning in the United States: The hurt could last a lifetime. Chicago, Illinois: McKinsey & Company Public sector Practice.

[ref12] Durlak J. A. Domitrovich C. E. Weissberg R. P. Gullotta T. P. (Eds.). (2015). Handbook of social and emotional learning: Research and practice. New York: Guilford Publications.

[ref210] Evans B. (2016). Paco—applying computational methods to scale qualitative methods. EPIC. 2016, 348–368.

[ref13] Fortus D. Krajcik J. (2012). Curriculum coherence and learning progressions. Secon International Handbook of Science Education. Berlin: Springer 783–798.

[ref14] Fredricks J. McClolskey W. (2012). “The measurement of student engagement: a comparative analysis of various methods and student self-report instruments,” in Handbook of Research on Student Engagement. eds. Christenson S. Reschly A. Wylie C. (MA: Springer), 7–37.

[ref15] Grabau L. J. Ma X. (2017). Science engagement and science achievement in the context of science instruction: a multilevel analysis of US students and schools. Int. J. Sci. Educ. 39, 1045–1068. doi: 10.1080/09500693.2017.1313468

[ref16] Hammerstein S. König C. Dreisörner T. Frey A. (2021). Effects of COVID-19-related school closures on student achievement-a systematic review. Front. Psychol. 12:289. doi: 10.3389/fpsyg.2021.746289, PMID: 34603162 PMC8481663

[ref17] Hektner J. Schmidt J. Csikszentmihalyi M. (2007). Experience sampling method: Measuring the quality of everyday life. Thousand Oakes, CA: Sage Publications.

[ref18] Immordino-Yang M. (2015). Emotions, learning and the brain: Exploring the educational implications of affective neuroscience. New York: W.W. Norton.

[ref19] Jagers R. J. Rivas-Drake D. Borowski T., (2018). Equity & social and emotional learning: a cultural analysis. Frameworks Briefs (November). Available at: https://casel.org/wp-content/uploads/2020/04/equity-and-SEL-.pdf

[ref21] Kraft M. (2020). Interpreting effect sizes of education interventions. Educ. Res. 49, 241–253. doi: 10.3102/0013189X20912798

[ref22] Krajcik J. Schneider B. (Eds.) (2021). Science education through multiple literacies: Project-based learning in elementary school. MA: Harvard Education Press.

[ref23] Krajcik J. Schneider B. Miller E. Chen I. Bradford L. Baker Q. (in press). Assessing the effect of the project-based learning on science learning in elementary schools. Am. Educ. Res. J.

[ref24] Krajcik J. Schneider B. Miller E. Chen I. Bradford L. Bartz K. (2021). Assessing the effect of the project-based learning on science learning in elementary schools. Final Technical Report to Lucas Education Research

[ref26] Krapp A. Prenzel M. (2011). Research on interest in science: theories, methods, and findings. Int. J. Sci. Educ. 33, 27–50. doi: 10.1080/09500693.2010.518645

[ref27] Lavonen J. Juuti K. Uitto A. Meisalo V. Byman R. (2005). Attractiveness of science education in the Finnish comprehensive school. Helsinki, Finland: Technology Industries of Finland.

[ref28] Lee C. Nasir N. Pea R. de Royston M. (2019). Introduction Reconceptualizing learning: a critical ask for knowledge building and technology. In Nasir N. S. Lee C. D. Peas R. de Royston M. M. (Eds.) Handbook of the cultural foundations of learning. (United Kingdom: Rutledge), 17–35.

[ref29] Lerner R. M. Steinberg L. (Eds.). (2009).Handbook of adolescent Pshycology. (3rd ed.). United States: John Wiley & Sons Inc.

[ref30] Miller E. C. Krajcik J. S. (2019). Promoting deep learning through project-based learning: a design problem. Disciplinary and Interdisciplinary Science Education Research 1, 1–10. doi: 10.1186/s43031-019-0009-6 PMC8325995 38624892

[ref31] Moeller J. Spicer J. Salmela-Aro K. Schneider B. (2017). “Advances in the research on situation-specific and contextual aspects of student engagement,” in Pathways to adulthood, educational opportunities, motivation, and attainment in times of social change. eds. Schoon I. Silbereisen R. K. (London: UCL IOE Press), 119–136.

[ref32] National Academies of Sciences. (2021). Science and engineering in preschool through elementary grades: The brilliance of children and strengths of educator’s. United States: National Academies Press.

[ref33] National Academis of Sciences. (2018). How People Learn II: Learners, Context, and Cultures. Washington, DC: National Academies Press.

[ref34] National Assessment of Educational Progress (2021). Results From the 2019 Science Assessment. U.S. Department of Education and Institute of Education Sciences.

[ref35] National Research Council. (1999). How People Learn: Brain, Mind, Experience, and School. United States: National Academies Press.

[ref36] National Research Council. (2012). A framework for K–12 science education: Practices, crosscutting concepts, and core ideas. United States: National Academies Press.

[ref38] Organisation for Economic Co-operation and Development. (2020). Science performance (PISA)—Indicator. Available at: https://data.oecd.org/pisa/science-performance-pisa.htm

[ref39] Peele-Eady T. B. Moje E. B. (2020). “Communities as contexts for learning,” in Handbook of the Cultural Foundations of Learning. eds. N. S. Nasir, C. D. Lee, R. Peas and M. M. de Royston (United Kingdom: Routledge), 230–246.

[ref40] Reniers R. L. E. P. Murphy L. Lin A. Bartolomé S. P. Wood S. J. (2016). Risk perception and risk-taking behaviour during adolescence: the influence of personality and gender. PLoS One 11:e0153842. doi: 10.1371/journal.pone.0153842, PMID: 27100081 PMC4839773

[ref41] Renninger K. A. Hidi S. E. (2020). To level the playing field, develop interest. Policy Insights Behav. Brain Sci. 7, 10–18. doi: 10.1177/2372732219864705

[ref42] Rosado-May F. J. Urrieta L. Jr. Dayton A. Rogoff B. (2020). “Innovation as a key feature of indigenous ways of learning: individuals and communities generating knowledge 1” in Handbook of the cultural foundations of learning. eds N. S. Nasir, C. D. Lee, R. Peas and M. M. de Royston (United Kingdom: Routledge), 79–96.

[ref43] Salmela-Aro K. Moeller J. Schneider B. Spicer J. Lavonen J. (2016). Integrating the light and dark sides of student engagement with person-oriented and situation-specific approaches. Learn. Instr. 43, 61–70. doi: 10.1016/j.learninstruc.2016.01.001

[ref45] Schneider B. Krajcik J. Lavonen J. Salmela-Aro K. (2020). Learning science: The value of crafting engagement in science environments. New Haven: Yale University Press. Also published in Chinese by, Educational Science Publishing House Limited, 2021.

[ref46] Schneider B. Krajcik J. Lavonen J. Salmela-Aro K. Broda M. Spicer J. (2016). Investigating optimal learning moments in U.S. and Finnish science classes. J. Res. Sci. Teach. 53, 400–421. doi: 10.1002/tea.21306

[ref47] Schneider B. Krajcik J. Lavonen J. Salmela-Aro K. Klager C. Bradford L. (2022). Improving science achievement—is it possible? Educ. Res. 51, 109–121. doi: 10.3102/0013189X211067742

[ref48] Shernoff D. Csikszentmihalyi M. Schneider B. Shernoff E. (2003). Student engagement in high school classrooms from the perspective of flow theory. Sch. Psychol. Q. 18, 158–176. doi: 10.1521/scpq.18.2.158.21860

[ref49] Shumow L. Schmidt J. A. (2014). Enhancing Adolescents’ Motivation for Science: Research-Based Strategies for Teaching Male and Female Students. Thousand Oaks, CA: Corwin, A Sage Company.

